# Adapting legume crops to climate change using genomic approaches

**DOI:** 10.1111/pce.13203

**Published:** 2018-06-13

**Authors:** Mahsa Mousavi‐Derazmahalleh, Philipp E. Bayer, James K. Hane, Babu Valliyodan, Henry T. Nguyen, Matthew N. Nelson, William Erskine, Rajeev K. Varshney, Roberto Papa, David Edwards

**Affiliations:** ^1^ UWA School of Agriculture and Environment The University of Western Australia 35 Stirling Highway Crawley Western Australia 6009 Australia; ^2^ School of Biological Sciences The University of Western Australia 35 Stirling Highway Crawley Western Australia 6009 Australia; ^3^ CCDM Bioinformatics Centre for Crop Disease Management, Curtin University Bentley Western Australia 6102 Australia; ^4^ Division of Plant Sciences and National Center for Soybean Biotechnology University of Missouri Columbia MO 65211 USA; ^5^ Natural Capital and Plant Health Royal Botanic Gardens Kew, Wakehurst Place Ardingly West Sussex RH17 6TN UK; ^6^ Centre for Plant Genetics and Breeding The University of Western Australia 35 Stirling Highway Crawley Western Australia 6009 Australia; ^7^ The UWA Institute of Agriculture The University of Western Australia 35 Stirling Highway Perth Western Australia 6009 Australia; ^8^ International Crops Research Institute for the Semi‐Arid Tropics (ICRISAT) Patancheru 502 324 India; ^9^ Department of Agricultural, Food, and Environmental Sciences Università Politecnica delle Marche 60131 Ancona Italy

**Keywords:** climate change, genomics, legume

## Abstract

Our agricultural system and hence food security is threatened by combination of events, such as increasing population, the impacts of climate change, and the need to a more sustainable development. Evolutionary adaptation may help some species to overcome environmental changes through new selection pressures driven by climate change. However, success of evolutionary adaptation is dependent on various factors, one of which is the extent of genetic variation available within species. Genomic approaches provide an exceptional opportunity to identify genetic variation that can be employed in crop improvement programs. In this review, we illustrate some of the routinely used genomics‐based methods as well as recent breakthroughs, which facilitate assessment of genetic variation and discovery of adaptive genes in legumes. Although additional information is needed, the current utility of selection tools indicate a robust ability to utilize existing variation among legumes to address the challenges of climate uncertainty.

## Abstract 2

Agriculture is facing a combination of factors that threaten global food security. In this review, we discuss some of the routinely used genomics‐based methods as well as recent breakthroughs that can help adapt legume crops to climate change by facilitating assessment of genetic variation and discovery of adaptive genes.

## 1 INTRODUCTION

The legume family (Fabaceae, syn. Leguminosae) is the third largest family of angiosperms, comprising over 750 genera and 19,000 species ranging from small herbs to large trees. The Fabaceae family is traditionally divided into three subfamilies: the Caesalpinioideae, Mimosoideae, and Papilionoideae. In the recent major taxonomic revision of the legume family, six subfamilies are recognized: the Mimisoideae is now a distinct clade with the Caesalpinioideae, four new subfamilies are described (Cercidoideae, Detarioideae, Duparquetioideae, and Dialioideae), and the Papilionoideae is largely unchanged (Azani et al., 2017). The majority of important grain and forage legume species are members of various clades within the Papilionoideae. This includes cool‐season legumes, such as lentil (Lens culinaris), chickpea (Cicer arietinum), and faba bean (Vicia faba; hologalegina clade), and warm‐season legumes, such as soybean (Glycine max), common bean (Phaseolus vulgaris), cowpea (Vigna unguiculata; phaseoloid/millettioid clade), *Lupinus* (genistoid clade), and *Arachis* (aeschynomenoid/dalbergioid clade; Bitocchi, Rau, Bellucci, et al., 2017; Doyle & Luckow, 2003; Gepts et al., 2005).

After the cereals, legumes are the most agriculturally important crop family (Graham & Vance, 2003) as they have multiple uses, ranging from animal forage and aquaculture feed to human food. Legume grains are appreciated for their protein content, in particular among low‐income families or where people avoid eating meat for religious or ethical reasons (Young, Mudge, & Ellis, 2003; Zhu, Choi, Cook, & Shoemaker, 2005). Legumes contain substances beneficial to health such as folate, lignans, saponins, antioxidants, dietary fibre, and resistant starch, and have the potential to offer protection against some cancers (American Institute for Cancer Research, 2014), diabetes, and obesity (Dove et al., 2011). Due to their symbiotic nitrogen‐fixing characteristics, legumes have a crucial role in natural ecosystems as well as in sustainable farming through their contribution in crop rotations and increasing soil fertility in arid areas and where nitrogen is low (Zahran, 1999).

## 2 CLIMATE CHANGE EFFECT

Demand for agricultural products continues to rise due to the population growth and increased food consumption per capita. Land‐use change and climatic variations are intensifying competition for resources (land, water, and energy; Abberton et al., 2015; Gomiero, 2016). Climate change impacts several aspects of agricultural systems, from altering flowering phenology, water availability, soil fertility and erosion, increase in pathogen spread, and host susceptibility (Rosenzweig & Hillel, 1995) to more subtle shifts in plant distribution and biodiversity (Bakkenes, Alkemade, Ihle, Leemans, & Latour, 2002), and plant‐pollinator interactions (Bishop, Jones, O'Sullivan, & Potts, 2016).

The combined effects of climate change on our agricultural systems can cause crop failures worldwide and lead to food insecurity. The complex challenge is best tackled with a joint approach that highlights the need for an increase not only in productivity (i.e., yield) and diversity of our crops, but also efficiency (i.e., water, land, and nutrient use; Abberton et al., 2015).

## 3 ADAPTATION PRIORITY IN REGIONAL AREAS?

Although climate change is a global threat, its direction and severity is not spread equally across continents and even regions. For example, although Mediterranean countries in Europe will experience frequent droughts, northern Europe is expected to become more of a Mediterranean climate. In Asia, more floods are expected in countries such as Bangladesh, due to the increase in severity of monsoon rains, whereas some others may experience decline in precipitation. In Africa, rise in temperature is predicted to increase desertification (Hopkin, 2005). As reconfirmed by the Global Climate Risk Index analyses, less developed countries are most vulnerable to climate change risk (Kreft, Eckstein, & Melchior, 2017), and hence their agriculture and food security will be also negatively affected, such as in Sub‐Saharan Africa as reviewed by Kotir (2011). Although the fragile nature of these regions makes them of great priority for maintaining agricultural productivity, to develop effective plans and make the best use of funding, ecosystem integrity needs to be taken into the account as well. In this context, Hannah et al. (2013) introduced several regional and global adaptation priorities by modelling the changes in agricultural suitability of 15 major rainfed staple crops, as well as biodiversity changes of 1,263 bird species. However, concerning crop development and breeding, we suggest that identifying climate‐related changes in biodiversity of crop wild relatives (CWR) along with farming suitability provides a more holistic approach to develop priority schemes.

## 4 WHICH TRAITS ARE IMPORTANT AS A TARGET OF BREEDING?

Yield is often the primary target of breeding, however, domestication traits such as flowering time, alkaloid content, and pod indehiscence have been the long‐term targets of breeding experiments as they contribute to yield total and quality. The multifaceted significance of flowering time in ecological, evolutionary, and adaptation processes makes it unique among traits that affect plant fitness (Elzinga et al., 2007; Franks, 2015; Weller & Ortega, 2015). A global search on 116 Northern Hemisphere plant families, including several species of legume, found global phylogenetic signals in the direction and magnitude of flowering time shifts, led by selection under climate change (Rafferty & Nabity, 2017). However, explaining the variation among or within species, and whether these shifts are sufficient for survival, remains unclear (Visser & Both, 2005). Thus, unravelling the genetic basis of flowering time variation is of great importance for breeding purposes (Nelson, Berger, & Erskine, 2010).

Efforts to alleviate the impact of climate change have led to increased research into traits such as drought and heat tolerance, as well as biotic stresses (Abberton et al., 2015; Doebley, Gaut, & Smith, 2006; Gepts, 2010). Soil and water salinity, which have been exacerbated due to the climate change driven factors such as sea level rise and shifts in precipitation (Teh & Koh, 2016), are also one of the major restrictions in the production of crops including legumes (Russell, 1976; Shrivastava & Kumar, 2015).

Efficient capitalization of elevated CO_2_ levels are of importance as this affects not only the plants, but also the rhizosphere microbial community structure and interaction (Drigo, Kowalchuk, & Van Veen, 2008). In addition to these adaptive traits, climate change is also forcing us to breed for novel traits. For example, genes that limit the production of methane in ruminants are being sought in forages, such as subterranean clover (Trifolium subterraneum), to mitigate greenhouse gas emissions (Kaur, Appels, et al., 2017).

Applying theoretical advances made possible through genomics studies can have practical outcomes, such as enabling plant breeders to accelerate the domestication of promising wild species. For example, in south‐western Australia where the legume farming system was dominated by clover and annual medics (*Medicago* spp.) prior to the 1990s, it became possible to expand legume diversity by incorporating traits such as deeper root systems, acid‐soil tolerant root nodule symbiosis, and pest and disease tolerance, and eight species of legumes were newly domesticated including Ornithopus sativus, Biserrula pelecinus, Trifolium glanduliferum, Trifolium dasyurum, Trifolium spumosum, Trifolium purpureum, *Medicago sphaerocarpos* (Nichols et al., 2007; Nichols, Loi, Nutt, Snowball, & Revell, 2010), and Melilotus siculus (common name messina; Rogers et al., 2011).

## 5 UNDERSTANDING GENETIC VARIATION

Knowledge of the extent and distribution of genetic diversity is essential for the efficient use of these resources in plant breeding programs. In order to understand the adaptation process, we must enhance our knowledge of mutations, genetic diversity of adaptive traits, phenotypic effects of genetic variants, and the interaction between the environment and genetic variation (Wright & Gaut, 2005).

Darwin (1859) considered domestication as a model of adaptation from which the nature of variation and selection could be inferred. Domestication of plants has been crucial to the development of human civilization by enabling an abundance of food (Diamond, 2002). However, as a consequence of this human intervention and historic population bottlenecks associated with it, the genetic diversity of most domesticated crops has been vastly reduced compared with their wild progenitors (Diamond, 2002; Gepts, 2010; Glémin & Bataillon, 2009). Reduction in genetic diversity of cultivated legumes compared with their wild relatives and ancestors has been discussed for different plants such as common bean (Bellucci et al., 2014; Bitocchi et al., 2013; Gepts, 1990), soybean (Hyten et al., 2006; Lam et al., 2010), narrow‐leafed lupin (Lupinus angustifolius; Berger, Buirchell, Luckett, & Nelson, 2012), and chickpea (Varshney et al., 2013). Interestingly, a study illustrated that reduction in genetic diversity as a result of domestication could go beyond the plant itself, as lower sequence variation was observed in rhizobia from domesticated chickpea compared with those from the wild type, which may suggest the potential negative impact of chickpea domestication on symbiosis (Kim et al., 2014). Shifts in genetic variation as a result of domestication, crop expansion, and breeding highlight the need for conserving and management of genetic resources for future breeding attempts.

## 6 RESOURCES AVAILABLE IN LEGUMES

### 6.1 Germplasm collections

Germplasm collections are the cornerstone of genetic resource conservation and management. Preserving wild plant populations in their natural habitat *in situ* will not only conserve diversity, but also ensures the extension of evolutionary processes that could lead to adaptive traits and new genetic and genotypic diversity for a wide range of species (Hawkes, 1991; National Research Council, 1993). The importance of *in situ* conservation of wild populations in particular for preservation of threatened Mediterranean legume genus such as *Lens*, *Lupinus*, and *Cicer*, due to the loss of natural habitats, ecosystems, and genetic diversity have been discussed and demonstrated (Maxted & Bennett, 2001; Walter & Gillett, 1998). However, there are currently few case studies of *in situ* conservation in legumes for wild species (Ajlouni, El‐Oqlah, Al‐Ghzawi, Al‐Tawaha, & Amri, 2010). However, it is also important to consider the *in situ*/on farm conservation of landraces and heterogeneous populations as a crucial aspect of the conservation of crop germplasm (Brush, 2000).

In the early 20th century, Nikolai I. Vavilov was among the first to recognize the significance of collecting and preserving plant materials *ex situ*. Vavilov's scientific expeditions resulted in conservation of genetic resources across different plant species including legume family members, such as white lupin (Lupinus albus), mung bean (Vigna radiata), chickpea, lentil, and pea (Pisum sativum; Kurlovich et al., 2000). Today, the extent of legume *ex situ* germplasm collections stands second only to the cereals, with a total of 1,041,345 accessions, out of which common bean represents the biggest group with 261,968, followed by tepary bean (Phaseolus acutifolius), scarlet runner bean (Phaseolus coccineus), lima bean (Phaseolus lunatus), and soybean, which collectively represent 156,849 accessions (Smýkal et al., 2015). For detailed information on the current number of accessions in legume germplasm collections, see Smýkal et al. (2015). These germplasm collections provide researchers with a great source of genetic variability that could be utilized in breeding for climate resilient crops (Hawkes, 1991).

### 6.2 Molecular markers and whole‐genome resequencing

In the last few decades, innovations in genomics‐based techniques and platforms have provided a wealth of genetic and genomics resources (Varshney, Graner, & Sorrells, 2005) that revolutionized research in both model and crop legumes. In particular, the increased application of molecular markers and reference genome sequences have had a substantial impact in accelerating progress in plant breeding and helping to incorporate new genetic diversity from germplasm resources.

Legume research has benefited widely from molecular markers of different types. For example, hybridization‐based markers, such as restriction fragment length polymorphism, were applied in legumes to develop linkage maps of common bean (Freyre et al., 1998), soybean (Keim, Diers, Olson, & Shoemaker, 1990), and narrow‐leafed lupin (Nelson et al., 2006); to assess genetic diversity in chickpea (Udupa, Sharma, Sharma, & Pai, 1993); and to identify the location of a gene for soybean mosaic virus resistance (Yu, Saghai Maroof, Buss, Maughan, & Tolin, 1994). These methods were subsequently replaced with polymerase chain reaction‐based markers, including both non‐specific markers (e.g., random amplified polymorphic DNA, amplified fragment length polymorphism (AFLP) markers) and locus specific markers (e.g., simple sequence repeats (SSR) and single nucleotide polymorphism (SNP) markers). Random amplified polymorphic DNA or AFLP markers have been employed to understand genetic structure of five wild lentil taxa (Ferguson, Newbury, Maxted, Ford‐Lloyd, & Robertson, 1998), construct a genetic linkage map in lentil (Eujayl, Baum, Powell, Erskine, & Pehu, 1998) and cowpea (Ouédraogo et al., 2002), and evaluate genetic diversity among *Lupinus* species (Talhinhas, Neves‐Martins, & Leitao, 2003). SSR markers (Gupta & Varshney, 2000) have been extensively used for constructing genetic maps in chickpea (Nayak et al., 2010), pigeonpea (Cajanus cajan; Bohra et al., 2011), and groundnut/peanut (Arachis hypogaea; Varshney et al., 2009). Development of SNP markers in common bean (Goretti et al., 2014), soybean (Wu et al., 2010), and narrow‐leafed lupin (Kamphuis et al., 2015) provide an opportunity for biodiversity conservation management programs and quantitative trait loci (QTL) fine mapping. The development of genetic linkage maps using SSR and AFLP markers in cultivated peanut (Hong et al., 2010) allowed a framework for further quantitative trait analysis and in lentil lead to find location of fusarium vascular wilt resistance (Hamwieh et al., 2005).

DNA sequencing technology has made major advances over the last decade, making many of the previous marker‐based systems redundant, and genome sequences are now available for many legume species, including cultivated soybean (Schmutz et al., 2009), Medicago truncatula (Young et al., 2011), *Lotus japonicus* (Sato et al., 2008), common bean (Schmutz et al., 2014; Vlasova et al., 2016), chickpea (Varshney et al., 2013), pigeonpea (Varshney et al., 2012), wild soybean (Kim et al., 2010), narrow‐leafed lupin (Hane et al., 2017), subterranean clover (Hirakawa et al., 2016; Kaur, Bayer, et al., 2017), and diploid ancestors (*Arachis duranensis* and *Arachis ipaensis*) of cultivated peanut (Bertioli et al., 2016). The availability of these resources provides an unprecedented opportunity for trait improvement through marker‐assisted evaluation of plant material (e.g., assessment of cultivars and genetic diversity), identification of QTL and gene discovery, marker‐assisted selection, and genomic selection.

## 7 FINDING ADAPTIVE GENES AND ADAPTIVE TRAITS

Currently, there are two general methods to identify genes and mechanisms related to important agronomic traits in plant species, known as “top‐down” and “bottom‐up.” The top‐down approach begins with a phenotype of interest followed by forward genetic analysis to identify candidate genes. Contrastingly, bottom‐up approaches use population genetic analyses to identify signatures of adaptation in a set of potentially adaptive genes, and then apply bioinformatics and reverse genetic tools to associate selected genes to a phenotype (Ross‐Ibarra, Morrell, & Gaut, 2007; Wright & Gaut, 2005).

### 7.1 Top‐down approach (linkage analysis)

#### 7.1.1 QTL mapping

Two popular genetic analyses used in the top‐down method are QTL and association or linkage disequilibrium (LD) mapping. QTL mapping is the more traditional approach and has been successful in identifying genomic regions associated with adaptive traits. For example, soil salinity is one of the major limitations for successful germination and plant growth in soybean (Essa, 2002), and several QTL mapping studies have identified loci conferring salinity tolerance (Do et al., 2017; Ha et al., 2013; Hamwieh et al., 2011; Lee et al., 2004).

QTLs have been widely used to identify genes corresponding to flowering time in various legumes such as soybean (Liu & Abe, 2009; Lu et al., 2015; Yamanaka et al., 2001; D. Zhang et al., 2013), mungbean (Isemura et al., 2012; Kajonphol, Sangsiri, Somta, Toojinda, & Srinives, 2012), pigeonpea (Kumawat et al., 2012), chickpea (Gaur, Samineni, Tripathi, Varshney, & Gowda, 2015), and common bean (Blair, Iriarte, & Beebe, 2006; Chavarro & Blair, 2010; González et al., 2016; Tar'an, Michaels, & Pauls, 2002), enabling genomics‐based breeding for adaptation traits.

Drought is a major limitation in the production of many legumes and has been targeted in various QTL studies to search for loci and genes conferring tolerance that has led to the breeding of crops with greater drought tolerance. A recent successful example is a study by Varshney et al. (2014) in chickpea, where they found several main effect and epistatic QTLs, among which, one QTL cluster was suggested as a “QTL‐hotspot,” a candidate genomic region for several drought tolerance and root traits in chickpea (Jaganathan et al., 2015; Kale et al., 2015). Later, applying a marker‐assisted backcrossing approach, this QTL‐hotspot was introgressed into a popular Indian chickpea variety (JG 11), which improved several root traits including rooting depth, root length density, and root dry weight (Varshney et al., 2016). This work was extended to several elite varieties in India and Africa (Thudi, Gaur, et al., 2014). Applying a QTL‐seq approach, that is, the identification of QTLs by whole genome resequencing from two bulked populations (Takagi et al., 2013), candidate genes for several traits under rainfed conditions (100‐seed weight, root/total plant dry weight) were rapidly identified in chickpea, and three genes have since been validated (Singh, Khan, Jaganathan, et al., 2016).

QTL analysis in cowpea led to the identification of five genomic regions accounting for 11.5–18.1% of phenotypic variation for heat tolerance (Lucas et al., 2013) as well as three loci associated with heat‐induced browning of seed coats (Pottorff et al., 2014). QTL mapping accompanied by synteny analysis revealed candidate genes for resistance to *Macrophomina phaseolina*, a fungal pathogen of cowpea (Muchero, Ehlers, Close, & Roberts, 2011). A recent study in pigeonpea used three different mapping populations and genotyping by sequencing to construct dense genetic maps that revealed 14 significant QTLs for resistance to fusarium wilt (Saxena, Singh, et al., 2017). Similarly, QTLs have been identified for sterility mosaic disease in pigeonpea (Saxena, Kale, et al., 2017). In addition, a QTL‐Seq approach has been used to map Fusarium wilt and sterility mosaic disease in pigeonpea (Singh, Khan, Saxena, et al., 2016). In wild lentil (*Lens ervoides*), a recent study by Bhadauria, Ramsay, Bett, and Banniza (2017) identified a total of 14 QTLs for resistance to *Colletotrichum lentis* (race 0 and 1) and *Stemphylium botryosum*. Several studies in common bean identified QTLs for resistance to different fungal (e.g., white mold, angular leaf spot, anthracnose, rust), bacterial (e.g., common bacterial blight, halo blight), and viral (e.g., bean common mosaic virus, bean common mosaic necrosis virus, beet curly top virus) pathogens (see Bitocchi, Rau, Rodriguez, & Murgia, 2016, as a review).

Although the identification of QTLs and candidate genes is relatively routine when a suitable population is available with good quality genotypic and phenotypic information, the translation of this information to the development of improved varieties can be challenging, and this method does have several limitations. For instance, developing mapping populations is difficult for some plants, such as those propagated vegetatively, perennial and polyploid species. In tetraploid alfalfa, which is a perennial species, availability of limited number of markers in a polyploid genome restricts the saturation of linkage maps. Furthermore, fewer recombination events are captured in a tetraploid population compared with the diploid, which affects the precision of linkage maps (Li & Brummer, 2012). Furthermore, QTL results are dependent on environment/experimental design and the allelic variations in parents of the experimental population (two parents in most studies). For example, identification of QTLs in regions with lower recombination rate, such as centromeric regions, will be more challenging. Additional drawbacks of QTL arise from what is known as the Beavis effect that is overestimation of phenotypic variances associated with QTL in a population of small size (Beavis, 1998; Korte & Farlow, 2013; Weinig & Schmitt, 2004; Xu, 2003).

Although identifying QTLs has its own challenges, narrowing down the QTL region to find the loci responsible for the trait of interest may not be easy. In addition to these challenges, lack of a thorough data management system for storing, combining, and reusing QTL data is an additional hurdle for efficient use of available information that could avoid doubling the efforts and expenses. Although this information is available for some major legumes through the legume information system (Dash et al., 2015), in cooperation with SoyBase (http://soybase.org) and PeanutBase (http://peanutbase.org), and cool season food legume (https://www.coolseasonfoodlegume.org), many of the minor legumes of great potential (such as *Lupinus* species) are not receiving enough attention in this regard.

An interesting study providing both opportunities and limitations of QTL is by Książkiewicz et al. (2017) in white lupin (Lupinus albus). Early flowering in white lupin was known to be controlled by the locus *brevis*, regulating vernalisation response, however, Książkiewicz et al. (2017) found multiple QTLs responsible for vernalisation responsiveness, yet the specific genes in these QTLs remain unknown. In addition, although overlapping QTLs were found in Australian and Polish experiments, identification of an additional small effect QTL in an Australian trial is a reflection that QTL results are environmental dependant, and hence, to capture the whole picture for a trait of interest, it is necessary to integrate and compare QTL data from different experiments.

#### 7.1.2 LD mapping (association mapping)

LD mapping has several benefits and can be considered as a complementary approach to QTL mapping. First, it may allow faster progress than QTL analyses as it does not always involve making experimental populations. Second and most importantly, LD can provide higher mapping resolution as it takes into the account the accumulation of historic recombination events (Korte & Farlow, 2013; Xu, Li, Yang, & Xu, 2017).

LD mapping can be classified into two types, including (a) broad genome‐wide studies seeking variation associated with phenotypic diversity and (b) narrower investigations attempting to identify causal genes and mutations in a small number of candidate genes within a specified genomic region (Ross‐Ibarra et al., 2007). Examples of where LD mapping has been applied for identification of both novel and previously characterized genes responsible for agronomic traits include genome‐wide association studies (GWAS) in model legume Medicago truncatula (Stanton‐Geddes et al., 2013), common bean (Kamfwa, Cichy, & Kelly, 2015; Moghaddam et al., 2016), and soybean (Contreras‐Soto et al., 2017; Zhou et al., 2015). GWAS has also proven to be successful in identifying candidate genes for ascochyta blight resistance (Li et al., 2017) and heat and drought tolerant loci in chickpea (Thudi, Upadhyaya, et al., 2014), and *Aphanomyces euteiches* resistance in Medicago truncatula (Bonhomme et al., 2014). Applying GWAS in a population comprising 292 pigeonpea accessions using data over several years enabled identification of association between candidate genes and traits, including 100‐seed weight, days to 50% flowering, and plant height (Varshney et al., 2017). Hoyos‐Villegas, Song, and Kelly (2017) investigated the genetic basis of variation for drought tolerance and related traits in a diversity panel including 96 Middle American genotypes of common bean, and the GWAS analysis allowed identification of significant marker‐trait associations for traits related to drought tolerance and candidate genes associated with wilting.

In cowpea, salinity has become an increasing threat to production, and Ravelombola et al. (2018) identified SNPs associated with salt tolerance at germination and seedling stages. These markers can be applied as a tool for selecting tolerant lines to be included in breeding programs of this crop. One of the most successful applications of GWAS is in peanut. Peanut is one of the important crop of the semi‐arid tropics, where climate change is posing a threat to crop productivity due to the increase in range of abiotic (e.g., drought and heat) and biotic stresses. Although the complex and tetraploid nature of the peanut genome makes QTL mapping studies a challenging task, GWAS enhances the chance of characterizing candidate genes for production related traits. A comprehensive study by Pandey et al. (2014) analysed marker‐trait associations for a wide range of economically important traits in peanut, such as yield components, oil components, drought, and disease tolerance. Several markers with significant allelic effects (>20% phenotypic variation) were identified for different traits such as pod yield, seed weight (under well‐watered and drought stress), oil content, and quality. Another GWAS study in 158 peanut accessions found a total of 51 SNPs associated with various traits including seed weight and pod weight, and identified candidate genes related to the domestication of peanut (Zhang et al., 2017), and this information will facilitate the genomic assisted breeding of peanut cultivars.

Despite the potential that LD mapping offers to identify adaptive genes, the tendency for spurious association, that is, false association with genomic regions, missing genotypes, identification of small effect variants, and genetic heterogeneity remain as limitations (Korte & Farlow, 2013). Another limiting factor of LD mapping is that resolution is dependent on the rate of LD decay, so using wild relatives of crops could serve as a better foundation.

In our opinion, because GWAS requires extensive phenotypic and genotypic information, it might be more usefully applied for major legume crops, where resources might already be available and the development of the future resources might be of interest of a wider research community. Additionally, accessing and integrating GWAS results from various studies is currently a cumbersome task due to the lack of a dedicated GWAS database in legumes that would enable cross referencing of resources from different experiments. The development of such a database would greatly benefit legume adaptation research.

#### 7.1.3 Genome‐environment association mapping

GWAS has been applied in genome‐environment association mapping to provide a new avenue to identify climate‐adaptive genetic loci and the genetic basis of local adaptation (Hancock et al., 2011), assuming “association between conditionally adaptive mutations and the environmental conditions with which they interact” (Turner, Bourne, Von Wettberg, Hu, & Nuzhdin, 2010 p. 262). Hence, genome‐environment associations along with genome‐phenotype associations can be applied to efficiently select for climate resilience traits (Lasky et al., 2015). In Arabidopsis lyrata, the association between polymorphisms and soil type was shown to be enriched in some functional annotation terms such as metal ion transmembrane transporter activity, providing novel candidate genes for soil adaptation (Turner et al., 2010).

In legumes, genome‐environment association analyses have been applied in Medicago truncatula, which identified candidate genes associated with adaptation to annual mean temperature and precipitation in the wettest month, and isothermality (Yoder et al., 2014). A recent study in narrow‐leafed lupin investigated the association between SNPs and climatic gradients and found significant associations between some SNPs with annual mean temperature and precipitation (Mousavi‐Derazmahalleh et al., 2018). Although availability of genotyping by sequencing data along with georeferenced genetic material makes genome‐environment association mapping an interesting avenue to explore, such analyses are most useful based on collections made directly from the wild habitat, ensuring good correspondence between climatic records and collection site. Hence, they may be less informative for legumes that have been domesticated a long time ago and diverged substantially from their wild ancestors. For example, in chickpea, which is one of the oldest domesticated legumes, hybrids from crosses between domesticated chickpea with *Cicer echinospermum*, a wild relative that is believed to have contributed gene flow to cultivated chickpea, are infertile (Ladizinsky & Adler, 1976).

### 7.2 Bottom‐up approach (population genomics)

The limitations of the QTL and LD mapping methods highlight the need for a complementary approach. Molecular population genetics, which forms the basis of bottom‐up approaches, appears to be promising for advancing our knowledge of the molecular signature of adaptation (Wright & Gaut, 2005).

Population genomics studies in Medicago truncatula demonstrated local adaptation of Tunisian populations to soil salinity, and revealed candidate genes with regulatory roles in abscisic and jasmonic acid signalling as well as genes associated with biotic stress and flowering time (Friesen et al., 2010; Friesen et al., 2014). A genome‐wide study of artificial selection in soybean revealed candidate genes for some domestication traits such as seed‐coat colour, growth habit, flowering time, and seed size (Li et al., 2013). Another study in soybean identified 159 putative domestication sweep accounting for 4.9% of the genome, containing 4,414 genes (Valliyodan et al., 2016). Recently, comparison of results of four different studies of varying sizes, data types, and methodologies (Bellucci et al., 2014; Bitocchi, Rau, Benazzo, et al., 2017; Rodriguez et al., 2016; Schmutz et al., 2014), all based on population genomics approaches to search for signatures of selection during common bean domestication, provided evidence of domestication candidate genes for four genes (i.e., *AN‐Pv33*, *AN‐Pv69*, *AN‐DNAJ*, and *Leg223*). Investigation of these genes highlighted their involvement in plant resistance/ tolerance to biotic and abiotic stresses, including heat, drought, and salinity (Bitocchi, Rau, Benazzo, et al., 2017).

Population genomics has great potential for identifying candidate genes harbouring adaptive mutations. However, careful consideration must be taken to exclude demographic effects such as population size and structure, which could bias the results by increasing the statistical variance applied to detect the selection signature. In addition, methods that consider demography may still not be able to detect recent selective sweeps (Nielsen, 2005). An example of where this can be seen is in narrow‐leafed lupin, a domesticated crop of the 21st century. We have recently assessed narrow‐leafed for signatures of selective sweeps at several domestication loci, including flowering time, pod‐dehiscence, alkaloid, and so forth, which were expected to show a signature of selection. However, the expected signal was only found at the flowering time locus, *Ku* (unpublished data). This can be explained in light of a recent study by Mousavi‐Derazmahalleh et al. (2018), who has shown that due to the local adaptation, the *Ku* locus in narrow‐leafed lupin has been under selection prior to the domestication of this crop. Additionally, the lack of selection evidence near other domestication genes could be illuminated by the strong population bottleneck during recent domestication of narrow‐leafed lupin (Berger et al., 2012).

Lastly, although interpreting patterns and distribution of selections in genomic regions can pinpoint the location of genes under adaptive selection, precise genome functional annotation for organisms are necessary to allow prediction of gene functions and their role in climatic adaptation.

## 8 GENERATING NOVEL DIVERSITY THROUGH CLASSICAL MUTAGENESIS

**Figure 1:**
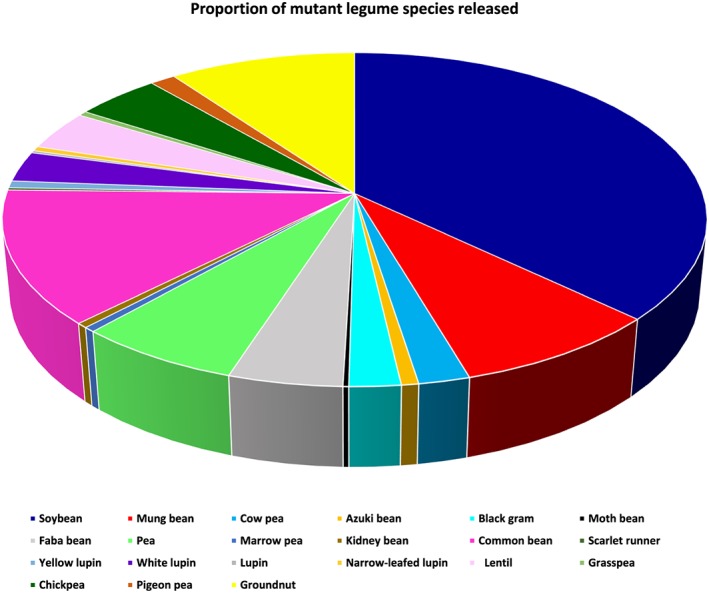
Proportion of mutant legume species released officially or commercially according to FAO/IAEA Mutant Variety Database as of October 31, 2017

Broadening the genetic base of crops through induced mutations has become a common practice for generating genetic variability for use in crop improvement programs (Sikora, Chawade, Larsson, Olsson, & Olsson, 2011). Both radiation (including X and gamma rays) and chemical‐based mutations (such as ethyl methane sulphonate and methyl nitrosourea) have been widely applied in legumes. More than 442 mutant varieties of legumes have been released officially or commercially worldwide according to the FAO/IAEA Mutant Variety Database (IAEA/FAO, 2017), with soybean accessions dominating the list, followed by common bean and groundnut (Figure 1). A wide variety of improved attributes have been associated with these mutants, including higher yield, resistance to virus and fungal diseases, early maturity, and tolerance to drought (IAEA/FAO, 2017). Mutagenesis breeding has introduced new genetic variation for breeding programs and has had a major impact on novel traits. For a comprehensive review on plant mutagenesis methods, please see Sikora et al. (2011).

## 9 OPPORTUNITIES

### 9.1 Epigenetic variation for crop improvement

In order to fully understand adaptive evolution, we should understand all possible changes leading to the adaptation. Therefore, whilst focusing on the role of genomics for producing climate‐ready crops, it is worthwhile to take into consideration the phenotypic alterations as a result of epigenetics variation. Epigenetics refers to mechanisms such as modification of DNA methylation and histones, and noncoding RNAs, which do not alter DNA sequences but could affect gene expression and trait phenotypes (Springer & Schmitz, 2017). Research in Arabidopsis thaliana has shown substantial heritable DNA methylation variation for several plant traits and plasticity, such as root allocation and drought tolerance, as well as the act of natural selection on some of the variation, such as plant biomass and height (Y.Y. Zhang, Fischer, Colot, & Bossdorf, 2013). A recent study by Song, Zhang, Stelly, and Chen (2017) revealed the contribution of methylated genes in the domestication of cotton, through induction of photoperiodic flowering due to the over expression of a photoperiodic regulating gene (*COL2*) after its demethylation. Although these studies provide excellent examples of the potential of epigenetics for identification of new sources of variation that can be applied in crop improvement, it is clear that genomics and epigenomics are not commonly integrated. Addressing this gap and combining forces between fields could lead to significant advances in breeding climate‐change ready crops, including legumes.

### 9.2 High throughput phenotyping

Recent decades witnessed a tremendous progress in DNA sequencing technologies; however, successful crop improvement plans are also dependant on accurately measuring plant traits to identify genetic loci associated with traits. Along with innovative and high throughout phenotyping strategies (such as near‐infrared spectroscopy on agricultural harvesters and spectral reflectance of plant canopy), analysis can be extended to the molecular phenotype using transcriptomic, metabolomics, and proteomic approaches (Beleggia et al., 2016; Bitocchi, Rau, Benazzo, et al., 2017). Together, these will improve our capacity to explore the phenotypic space from large multilocation field trials (Fahlgren, Gehan, & Baxter, 2015; Montes, Melchinger, & Reif, 2007). A recent study on a large population of rice (consists of 1,568 samples), using both field‐based high throughput phenotyping (HTP) and manual phenotyping, confirmed the efficiency and accuracy of HTP in detecting QTLs associated with grain yield and yield components (Tanger et al., 2017). Of relevance is also the development of a high‐throughput phenotyping system to study root systems (Gioia et al., 2017), and integrating HTP methods with high‐throughput genotyping hold a potential to unravel the genetic basis of complex traits, such as heat and drought tolerance.

### 9.3 Predictive modelling

Exploring collections of CWR from regions that are likely to be enriched for target traits (e.g., warm, dry areas for heat and drought tolerance) ensure this available genetic diversity can be identified and harnessed when needed (Mousavi‐Derazmahalleh et al., 2018; Phillips, Asdal, Magos Brehm, Rasmussen, & Maxted, 2016). Predictive species distribution modelling can play an important role in inferring the full geographical range of species' natural habitat. Furthermore, the availability of ecogeographic land characterization (ELC) maps allow the identification of ecogeographical zones representing adaptive scenarios for plants, and can assist breeders to find genotypes under adaptive forces. The application of ELC maps to explain seed weight variation in a range of different plant species, including four legumes (Lupinus angustifolius L., *Vicia sativa* L., *Pisum sativum* L., and Phaseolus vulgaris L.), was useful in revealing favourable and marginal environments (Parra‐Quijano, Iriondo, & Torres, 2012). Applying predictive species distribution modelling accompanied by ELC maps can pave the way to conserve CWR *in situ* and *ex situ*, ensuring that a broad range of genetic variation has been captured (Parra‐Quijano et al., 2012; Phillips et al., 2016).

### 9.4 Pangenomes

Although the availability of reference genomes has greatly assisted plant genetics research and breeding, these reference genomes capture only a portion of diversity present in the species. A solution is the development of pangenome assemblies that more comprehensively capture sequence and structural diversity in a species. In legumes, pangenomes have been constructed for soybean (Lam et al., 2010; Li et al., 2014) and Medicago truncatula (Zhou et al., 2017). A pangenome of soybean based on seven accessions of wild soybean suggested faster evolution and greater variability in dispensable genes compared with the core genes, which may be associated with adaptation to biotic and abiotic stresses (Li et al., 2014).

Pangenome construction takes into the account the structural variations, and so they capture the genetic variation of the species more comprehensively rather than a single reference genome. They also enable comprehensive identification of SNP variation. This simplifies discovery of rare variants, which might be associated with QTLs for agronomic traits. Pangenomes also allow the differentiation between SNPs occurring in core (present in all individuals of the species) and variable (present in a subset of individuals) genomes, the latter of which was found in several studies to influence adaptation to biotic and abiotic stresses (Hurgobin & Edwards, 2017). These resources can be of value for legume breeding, based on novel gene identification and discovery of nucleotide diversity that enables molecular marker design for introgression of previously untapped genes into crop improvement programs.

Considering the fact that the selection of appropriate individuals with enough variation is an important element to a successful pangenome study, we suggest that pangenomes may be smaller in size in crops such as narrow‐leafed lupin, which went through severe genetic bottleneck during its recent domestication (Berger et al., 2012). In addition, pangenome construction requires extensive sequence data and computational resources, and its quality is dependent on the assembly precision. This makes the development of pangenomes challenging in the case of crops with complex and large repetitive genomes such as pea (Macas, Neumann, & Navrátilová, 2007), as well as polyploid genomes such as tetraploid alfalfa (Medicago sativa).

### 9.5 Genome editing

A relatively new technology for mutagenesis is the clustered regularly interspaced short palindromic repeat (CRISPR)/CRISPR‐Cas9 system. Originally discovered as bacteria's adaptive immune system, CRISPRs' repeat‐spacer‐repeat sequence pattern was found to be involved in an RNA intereference‐like mechanism that can identify and cut foreign DNA. Genome editing modification by CRISPR uses a guide RNA that is complementary to a target gene, induces double‐strand breaks usually by a Cas9 nuclease, followed by a non‐homologous end joining or homology‐directed repair mechanism (Jinek et al., 2012; Scheben & Edwards, 2017; Xiong, Ding, & Li, 2015).

CRISPR/Cas9 has been applied in model legume plants. Michno et al. (2015) designed a web‐tool that can rapidly find numerous potential CRISPR/Cas9 target sites, as well as a soybean codon‐optimized CRISPR/Cas9 platform that induced targeted gene mutation in somatic cells of both Glycine max and Medicago truncatula by root hair transformation. A recent study in Medicago truncatula targeted the MtPDS gene involved in carotenoid biosynthesis, which was successfully disrupted by an optimized agrobacterium‐delivered CRISPR/Cas9 platform (Meng et al., 2017). The above examples in addition to the availability of high quality reference genomes highlight the potential of CRISPR/Cas9 beyond the model legumes.

CRISPR has become a popular choice for genome editing in plants due to ease of use, lower cost, and ability to edit multiple targets that enables genes pyramiding into a new cultivar within a single generation. Furthermore, unlike traditional breeding methods, CRISPR is not restrained by the existing diversity as it can directly introduce new mutations. This would be beneficial, especially for crops that have low variation for traits of interest and where natural variations cannot be find in nature. In addition, although crossing or backcrossing methods may result in introduction of deleterious alleles, genome editing is unlikely to cause this issue.

Although CRISPR offers an unprecedented opportunity for crop improvement, the starting point of a CRISPR approach is the comprehensive knowledge of the target gene(s), its function and regulation. This may restrict the use of CRISPR in crops that have limited information of genes involved in adaptation processes. Nevertheless, the decreasing cost of genome sequencing accompanied by the increase in precision of genome assemblies and functional annotation could improve gene prediction, though it should be emphasized that experimental characterization of genes of interest remains necessary for successful results. For a comprehensive review, see Scheben, Wolter, Batley, Puchta, and Edwards (2017).

## 10 CONCLUSIONS AND PERSPECTIVES

Legumes hold great promise to mitigate the effect of climate change through their contribution in sustainable farming, capitalization of elevated level of CO2, and broadening the crop base, which is currently dominated by a small number of major crops, mainly from the cereal family. In addition, enormous progress has been made in legumes to identify novel alleles for adaptive traits. However, deployment of these findings in applied breeding remain a major limitation to release climate‐ready cultivars. As stated by Gready (2014), disruptive thinking and technologies are required to take advantage of best of the old and the new. We believe availability of genome editing tools such as CRISPR provide an excellent example of this. The full potential of legume crops remains yet to be explored, with genomics as a powerful enabling tool. Choice of approaches to create new cultivars is dependent on various factors such as plant information, availability of genomic, and phenotypic resources, nature of traits (simple or polygenic) and countries' regulations. Traditional and modern breeding approaches contributed (and will contribute) in creating improved crop varieties. However, the urgency for crop improvement, driven by fast pace of climate change and rapid population growth, emphasize the need for thinking outside the box. CRISPRs allow to create novel cultivars with multiple genes only in one generation. This substantially speed up the process of creating crops adapted to the ever‐changing environment and ensures that agriculture can keep up with the velocity of climate change.

## CONFLICT OF INTEREST

None.

## AUTHOR CONTRIBUTIONS

MMD drafted the manuscript and prepared the figure, under supervision of DE. DE, RP, RKV, WE, MNN, HTN, VB, JKH, and PEB provided comments and revised the manuscript. All authors have read and approved the final manuscript.
